# Diamond thin films: giving biomedical applications a new shine

**DOI:** 10.1098/rsif.2017.0382

**Published:** 2017-09-20

**Authors:** P. A. Nistor, P. W. May

**Affiliations:** 1 Regenerative Medicine Laboratory, University of Bristol, Bristol BS8 1TD, UK; 2 School of Chemistry, University of Bristol, Bristol BS8 1TS, UK

**Keywords:** chemical vapour deposition diamond, cell culture, bioimplants, neuron growth, artificial neural networks, bone scaffolds

## Abstract

Progress made in the last two decades in chemical vapour deposition technology has enabled the production of inexpensive, high-quality coatings made from diamond to become a scientific and commercial reality. Two properties of diamond make it a highly desirable candidate material for biomedical applications: first, it is bioinert, meaning that there is minimal immune response when diamond is implanted into the body, and second, its electrical conductivity can be altered in a controlled manner, from insulating to near-metallic. *In vitro,* diamond can be used as a substrate upon which a range of biological cells can be cultured. *In vivo*, diamond thin films have been proposed as coatings for implants and prostheses. Here, we review a large body of data regarding the use of diamond substrates for *in vitro* cell culture. We also detail more recent work exploring diamond-coated implants with the main targets being bone and neural tissue. We conclude that diamond emerges as one of the major new biomaterials of the twenty-first century that could shape the way medical treatment will be performed, especially when invasive procedures are required.

## 1. Introduction

### 1.1. Diamond for *in vitro* and *in vivo* applications

Since the middle of the twentieth century, *in vitro* cell-culture techniques have made great advances in terms of popularity, volume, types of cells to which they are amenable and downstream applications. To culture living cells in the laboratory, a requirement is a surface that is cheap to manufacture, smooth and sterilizable, and which minimally affects the normal growth, lifetime and functionality of the chosen cells. The usual substrates of choice are either glass or an inert plastic, such as polytetrafluoroethylene (PTFE) or tissue-culture-grade polystyrene (TCP). Many different cell types can be readily and routinely cultured on such substrates. *In vitro* cell-culture tests are often used for prospective *in vivo* biologically active implant experiments. However, glass, PTFE and TCP differ significantly from the materials used for fabricating these devices. Maximum translation from *in vitro* to *in vivo* would be obtained if a material equally suited for both types of applications could be found.

### 1.2. Diamond films can be produced by chemical vapour deposition

One such substrate material that has recently been gaining in popularity is thin film diamond. At first glance, diamond may seem like an odd choice from which to make a biological plate [1,2], as diamond is often perceived as being used only for jewellery or cutting tools, and overly expensive as a cell-culture substrate. However, in the past 20 years, scientists have perfected a technique known as chemical vapour deposition (CVD) which allows thin coatings of pure crystalline diamond to be deposited onto a range of different materials in a cost-effective manner [3]. Many companies now supply CVD diamond commercially—indeed, a freestanding plate of diamond that is 1 cm^2^ in size by 0.5 mm thick costs only about $50 USD—hardly expensive, especially when considering that the plates can be easily cleaned, sterilized and reused. Moreover, many university research groups and commercial companies will supply thin coatings (a few µm) of diamond on a silicon wafer for a fraction of this cost, meaning that the diamond-coated substrate, far from being unaffordable, is actually cheaper than many of the reagents needed to grow the cells!

### 1.3. Diamond films are biocompatible

But what advantage does diamond offer? The first, and probably most important benefit is its high biocompatibility, a property confirmed by nearly a century of studies [4–10]. In early reports, very low toxicity was observed when diamond particles were injected as a suspension into live animals [5,11,12]. Subsequently, it was established that diamond films as a cell-culture substrate can successfully support a wide range of adherent cells. In this review, we will describe in detail the efforts made to optimize the cell-supportive properties of diamond films by modulating the crystal granularity, chemical surface termination (with major implications on hydrophilicity), as well as by patterning. Another crucial property of diamond is that it is bioinert, i.e. it does not trigger coagulation or inflammatory reactions. It should also be noted that, whereas most bioinert materials show poor cell adherence and *vice versa*, diamond is both bioinert and cells readily adhere to it [13–17].

### 1.4. Diamond films can be electrically conducting

The second advantage is that diamond electrical conductivity can be controlled by a process called doping (i.e. addition of tiny amounts of non-carbon elements, acting as electron donors or acceptors), with the conductivity varying from highly insulating to near-metallic, depending upon the amount of dopant added. Indeed, very heavily doped diamond even exhibits superconductivity at temperatures around 5 K [18,19]. Elements such as nitrogen and phosphorus have been used to dope diamond films [20]; however, except in the case of ultrananocrystalline diamond (UNCD) films (see later) [21], the resulting conductivity is usually too low to make these films useful for many electronic applications. The dopant of choice, therefore, is boron, an element widely used for routine doping of both silicon and diamond in semiconductor research. An electrically conducting diamond substrate allows electrical signals to be passed to and from the cells it supports. This is particularly useful in the case of neurons, potentially allowing for direct, two-way communication between supported cells and the underlying substrate. Electrically conductive diamond can also be used as an electrochemical electrode which is particularly useful for biosensor applications [22].

### 1.5. Diamond can be patterned and its surface chemically functionalized

**Figure 1.**
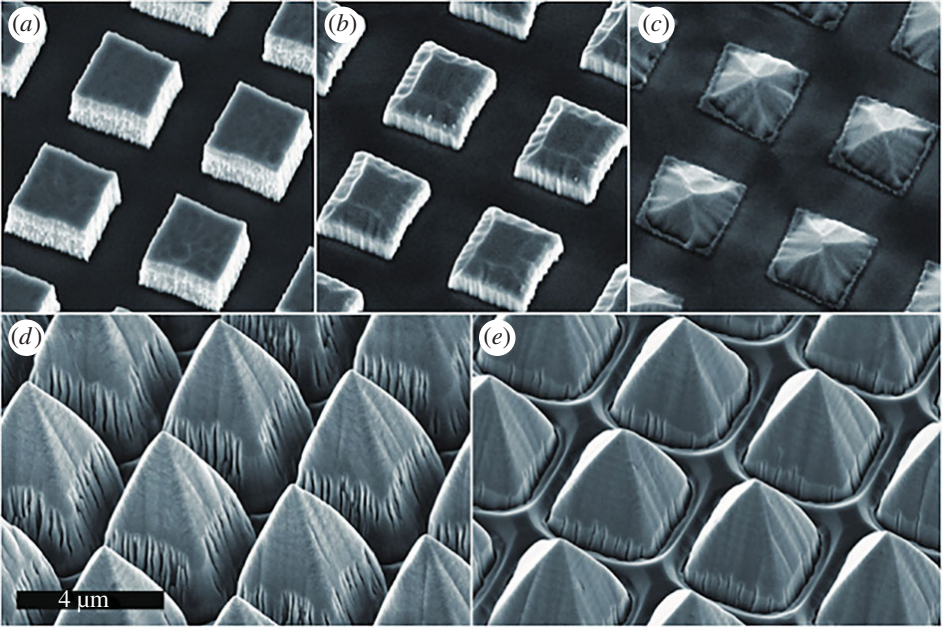
Examples of CVD diamond films patterned using dry etching methods. In (*a*–*e*), a variety of different etch processes and mask preparations have been used to define features with different sizes, shapes and sidewall slopes. Similar dry etch methods can be used to pattern diamond films into almost any desired shape, including needles, columns, pyramids, etc. Reproduced from [25] under CC-BY licence. (Online version in colour.)

A third advantage is that a diamond surface can be readily patterned and/or functionalized to define regions that sustain cell growth, and regions that do not. A wide range of methods have been used to pattern diamond, with pattern resolutions ranging from millimetres to micrometres [23,24]. Diamond films and freestanding substrates are compatible with the fabrication facilities used for making silicon microelectronics, and hence the standard photolithography and dry etching processes used for patterning Si can also be applied to diamond. Diamond etches readily in oxygen-based plasmas, with gaseous CO and CO_2_ as by-products. The slight complication is that a two-step etch process must be used [25–27], as the usual carbon-based photoresists etch away rapidly in oxygen plasmas, resulting in poor pattern resolution. To solve this, a thin (100 nm) non-erodible mask material, such as Ni or Al, is deposited onto the diamond prior to the standard photolithography process, which defines the features, and then this metal layer is dry etched in a suitable gaseous plasma or wet etched in acid, stopping at the diamond surface. The exposed diamond surface can then be etched in an oxygen-based plasma with the patterned metal acting as a ‘hard-mask’ (figure 1). This metal mask and any remaining photoresist can finally be removed using a simple acid wash.

Diamond can also be patterned by direct-write laser ablation [24]. This uses a high-power laser (usually an excimer or Nd:YAG) which is focused to a spot on the surface. The energy density at the laser focus spot is so great that a few atomic layers of the diamond surface are instantly vaporized. The amount of material removed with each laser pulse depends upon the laser power, wavelength and spot size. To define the features, either the substrate is moved under the static laser beam, or the laser beam is scanned across the surface using mirrors.

An alternative patterning strategy, which works well for deep (greater than 50 µm) features, involves depositing the diamond film onto a substrate, usually silicon, that has been pre-patterned using standard etch recipes into features that are the inverse of those required. This patterned substrate then acts like a mould, onto which the diamond film is conformally deposited. The substrate is then flipped upside down, and the Si chemically etched off, leaving behind a freestanding patterned diamond substrate [28].

Rather than pattern the diamond itself, a different approach is to pattern the layer of protein (usually laminin or poly-l-lysine) which is often applied to the surface of the diamond film to promote cell adhesion (see §4.3). This protein layer can be readily patterned using direct-write focused laser beams, or it can be stamped onto the surface using a micro-contact printing method [29]. Cells cultured on this substrate will generally only survive on the protein-coated areas, and will die on the uncoated areas.

In terms of functionalizing the diamond surface, a number of techniques are commonly used [30,31]. Oxidation is done either by short exposure to an O_2_ plasma, ozone treatment (via a high-power UV lamp), or by placing the diamond into a hot mixture of nitric and sulfuric acid for an hour. These treatments convert the C–H surface bonds into C–O bonds, thereby changing the surface properties from hydrophobic into hydrophilic. This process can be reversed by exposure of the oxidized film to a H_2_ plasma. Similarly, exposure to an NH_3_ plasma or a halogen-containing (e.g. Cl_2_, CF_4_, SF_6_) plasma will aminate (–NH_2_ groups) or halogenate (Cl or F) the surface, respectively. More complex ‘linker’ molecules can be tethered to the diamond surface using standard synthetic chemistry techniques such as Suzuki coupling and UV photo-addition [32]. The untethered end of these linker molecules, which are usually terminated with reactive groups like amines or carboxylic acids, can then be further bonded to molecules with specific (bio)chemical functionality, such as proteins, DNA strands, fluorophores and antibodies [33].

Despite all these technological advancements, diamond has still been rather slow to enter mainstream biomedical applications. Among potential reasons for this is a lack of awareness of doctors and medical investigators to regard diamond as a biomaterial. Often diamond research is presented only in diamond-themed conferences with little participation from the medical community. Furthermore, commercially available diamond films are rarely advertised for their biomedical applications. Also, medical researchers can be somewhat conservative when it comes to embracing new unproven materials, especially ones that are perceived as being niche or overly expensive. Often, when the awareness exists, doctors cite diamond's rigidity as a potential concern, which is a valid point if diamond were to be used either as large-area implants, or elongated implants in soft tissues such as muscles, but less of an issue if it is to be used as an electrode layer, fractions of a millimetre thick, at the end of a flexible biomaterial.

Regardless of the relatively slow uptake, the importance of diamond in medical research has been underscored in recent years by major collaborative research projects such as *Neurocare* (http://neurocare-project.eu/) and *DREAMS* (http://cordis.europa.eu/docs/publications/1235/123542961-6_en.pdf) in Europe, the *Bionic Eye* (http://www.nvri.org.au/pages/bionic-eye.html) in Australia and the *Argus® II Retinal Prosthesis System* developed jointly by Argonne National Labs and Second Sight Medical Products, Inc. (http://www.secondsight.com/) in the USA. These national or multinational projects brought together top institutions from their respective countries to investigate a potential role for diamond implants in state-of-the-art treatments designed to improve the quality of life of otherwise severely impaired patients.

In this review, we shall summarize the current data on the interaction between diamond and living organisms. We will provide a brief overview of the many experiments that have been performed to grow a range of mammalian cells on CVD diamond substrates and the methods employed. We will then focus specifically on potential clinical applications such as *in vitro* neuronal cells grown on diamond, and diamond brain implants, as well as diamond-based bone and joint implants. Finally, we shall discuss the advantages and disadvantages of diamond substrates for the various *in vitro* and *in vivo* applications, and speculate where this technology may lead.

## 2. Chemical vapour deposition diamond

### 2.1. The chemical vapour deposition process

Synthesis of diamond films by CVD techniques was first developed in the late 1980s, and since then it has matured into a commercial technology, with a number of companies worldwide (such as *Element Six* (UK) (http://www.e6cvd.com/), *IIa Technologies* (Singapore) (http://2atechnologies.com/), *Fraunhofer IAF* (Germany) (http://www.iaf.fraunhofer.de/), Advanced Diamond Technologies (Illinois, USA) (http://www.thindiamond.com/) or *Diamond Materials* (Germany) (http://www.diamond-materials.com/EN/index.htm), as well as others in Japan, India, China and Russia, now supplying CVD diamond films or plates at affordable prices. The CVD process involves the reaction of a mixture of carbon-containing gas (usually methane) and hydrogen in a low-pressure reactor, followed by the deposition of the carbon in the form of a crystalline diamond coating onto a suitable substrate material. The substrate can be a diamond, in which case the CVD process will increase its size. This is the basis for the burgeoning industry in CVD diamond gemstones destined for the jewellery market [34]. Alternatively, if the substrate is made from another material, such as silicon, then the diamond is deposited as a thin coating or film, which can be nanometres, micrometres or even millimetres thick, depending upon growth conditions and time. The diamond coating can remain attached to the substrate, or the substrate can be chemically or mechanically removed, producing a freestanding diamond plate.

### 2.2. Types of chemical vapour deposition diamond films

The ultimate material produced by CVD is single-crystal diamond (SCD), which is almost indistinguishable from gemstone material. However, the control of the deposition conditions necessary to produce large areas of SCD is not yet precise enough, and hence, at the moment, SCD substrates are limited to areas less than 10 × 10 mm and have a correspondingly high price. Currently, the most affordable CVD diamond films come in the form of a *polycrystalline* coating, which is a dense layer made up of many smaller diamond crystallites joined together by atomic-scale non-diamond (usually graphitic) grain boundaries. The size of the crystallites and the number of grain boundaries in the film determine its mechanical and electronic properties—as well as its bioproperties.

### 2.3. Polycrystalline chemical vapour deposition diamond films

**Figure 2.**
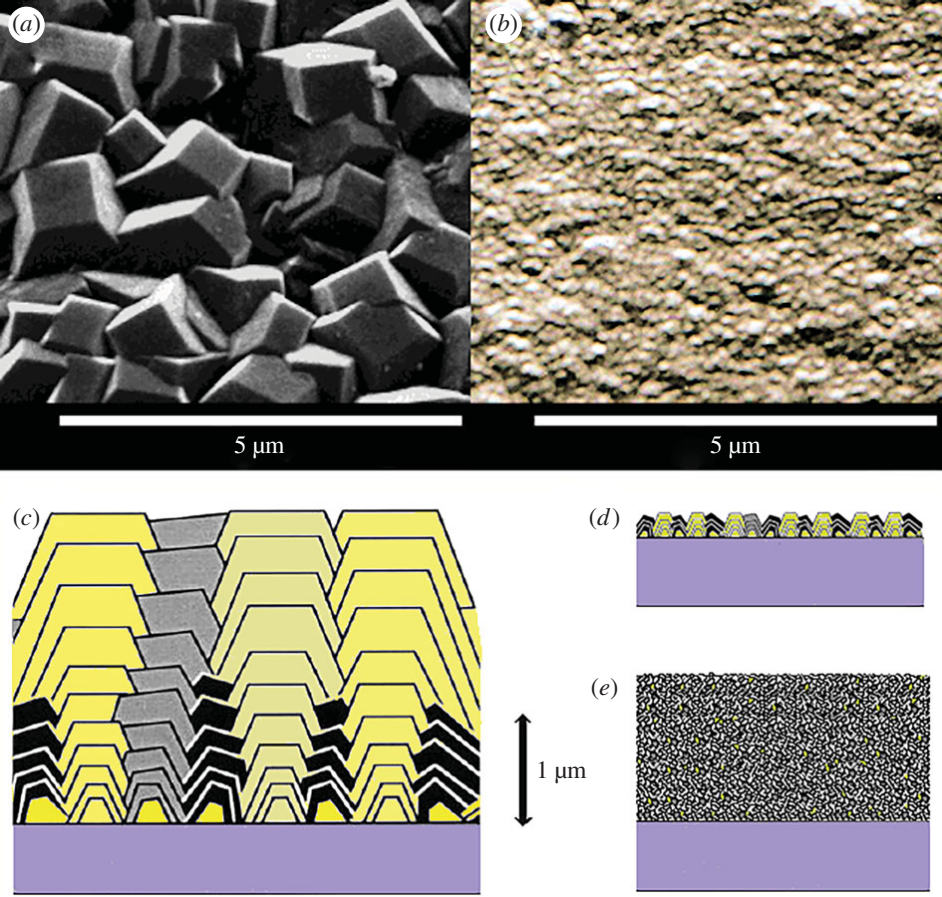
Electron micrograph images of top views of (*a*) MCD and (*b*) NCD films. On this magnification scale both SCD and UNCD would appear flat and featureless. Below are schematic representations of cross sections through (*c*) MCD with its characteristic columnar structure, (*d*) NCD deposited under MCD growth conditions but for only a short time such that the film remains facetted but is ≪ 1 µm thick, and (*e*) NCD grown with higher methane concentration such that the columnar structure is lost and the film is smoother with smaller (approx. 100 nm), more rounded grains. On this scale, UNCD would appear very similar, except that the grains would be even smaller (less than 10 nm), while the films appear smooth (r.m.s. roughness less than 5 nm) with thickness usually less than 1 µm. (Online version in colour.)

Polycrystalline CVD diamond films are usually categorized into three types based on the average size of the crystallites. First, there is *microcrystalline diamond* (MCD), where the grain sizes are approximately 0.5–100 µm in size. These films have relatively few grain boundaries and exhibit large, faceted crystals arranged in different orientations (figure 2). MCD films usually exhibit properties that are almost as good as that of SCD but the large crystallites produce a surface roughness on the micron scale, which is not amenable to some types of cell growth. By contrast, *nanocrystalline diamond* (NCD) films contain grain sizes in the range of 10–100 nm, and the crystallites are often rounded and much less facetted. Although cells have been cultured on a variety of diamond crystallite grain sizes, a number of groups have reported slightly improved results on NCD films [14,17,35–37]. Finally, UNCD films have grain sizes that are less than 10 nm, and grain boundaries containing amorphous or sp^2^ carbon that are almost as wide as the grains. UNCD is thus better described as a composite material of diamond and amorphous carbon material. Nevertheless, UNCD still retains sufficient diamond-like character to be useful for many applications, particularly those where a nanosmooth surface with a low coefficient of friction is important, such as mechanical pump seals or coatings for artificial hip/knee prostheses [38]. It is also the only type of diamond film where nitrogen, rather than boron, can be used as a useful dopant, producing highly conducting N-UNCD material [21].

### 2.4. Diamond-related materials

There are two other materials related to diamond which require mentioning. *Nanodiamond particles* (NDPs), sometimes also called ‘detonation nanodiamond’, are crystals of diamond that range in size from a few 100 nm down to 3 nm, and which can now be bought commercially as powders or as aqueous suspensions [39]. NDPs are receiving a lot of attention for their use as fluorescent biomarkers [40] or as vehicles for targeted drug delivery [41] and regenerative medicine [42]. By dipping a substrate into an NDP suspension (often with the aid of ultrasonic agitation and/or the use of charged polymers to help with electrostatic adhesion), a thin, near-continuous NDP coating forms, which can then be used for cell growth, or other applications [43]. The advantage of this approach is that it is very cheap, and allows bespoke NDP layers to be fabricated easily without the need for a CVD step. However, NDPs do not integrate into a compact layer and therefore they cannot be used to construct flat or three-dimensional electrically conducting tracks. Nevertheless, NDPs could be used in combination with CVD diamond films as an adhesion-promoting coating, or to alter the chemical properties of the diamond surface. There are reports [44] that using an NDP layer removes the requirements for a protein intermediate layer (see §4.3) that is usually used when CVD diamond substrates are used for cell growth [45]. Although the focus of this review is on diamond films and not NDPs, we shall nevertheless discuss NDPs in relation to their bioinert properties. This is because NDPs have a higher risk of being cytotoxic compared with thin films, and also because diamond-coated implants could release NDPs into the surrounding tissues, especially if used in areas of high friction, as would be the case for artificial cartilage surfaces.

The second material of note is *diamond-like carbon* (DLC), which should not be confused with CVD diamond. DLC is a hard, nanosmooth, amorphous carbon material deposited onto a substrate by physical bombardment, e.g. ion sputtering [46]. Although it can have up to 50% the properties (e.g. hardness) of diamond, and has a high degree of bioinertness, it is not crystalline, and may contain significant concentrations of elements such as H, N and O. DLC is actually a broad term covering a wide range of carbon materials composed of a variety of nanoscale diamond-like and graphite-like regions, and hence a wide distribution of properties. Many different DLC coatings have been evaluated as substrates for cell growth, and as materials for medical biocoatings for over 20 years [47–49], including protective coatings for hip replacement joints, non-stick inert coatings for artery stents and coatings for optics. DLC is a large subject, and not the focus of this review, which will concentrate on crystalline CVD diamond.

## 3. Diamond biocompatibility

### 3.1. Biocompatibility of nanodiamond particles

Diamond is one of the most chemically inert materials known. As a consequence, it is also ‘bioinert’—it does not engage in biochemical reactions with the internal environment of an organism, including the human body. The advantage of this is that implanted *in vivo* diamond-based devices offer the prospect of reduced immune response, with little or no inflammation, leading to device lifetimes that can presumably exceed that of the patient. However, there is a risk that nanosized NDPs could accumulate inside scavenger cells, which would be unable to metabolize them, leading to cell toxicity. Perhaps surprisingly, experiments performed in the first part of the twentieth century indicated that this might not be a problem. NDPs injected into natural cavities (e.g. the peritoneal cavity and lungs) or into the bloodstream of rats did not seem to have any observable detrimental effects [5,11,12]. More recent research confirmed that intra-peritoneal injection of fluorescent NDPs (up to a high dose of 75 mg kg^−1^) also had no observable toxic effects in rats [6]. Furthermore, *in vitro* data indicated that NDPs of various sizes (2–100 nm) can be uptaken by mammalian cells with minimal cytotoxicity and a low level of oxidative stress [7,50]. This is in contrast with other types of nanoparticles, which induce oxidative stress and activate an inflammatory gene profile upon internalization into human cells [51–53].

An adult organism is endowed with significant robustness and resistance to extrinsic (bio)chemical threats. This is, however, not the case of a developing organism, which could suffer major damage from otherwise benign challenges. Nevertheless, when NDPs were administered *in ovo* no adverse effect was observed on chicken embryo development [8].

### 3.2. Biocompatibility of diamond films

When considering diamond films and coatings as the outer layer of implantable devices, a key aspect of biocompatibility is the potential of the film (or any particulates that may arise from this as a result of device wear or failure) to activate the immune response. The first line of defence of the body consists of *neutrophils*. These cells constantly patrol the inner linings of the blood vessels in search of intruders. Upon discovering a lesion, they respond by adhering to the site and secreting granules (degranulation). The chemicals contained inside the granules activate tissue inflammation, generate reactive oxygen species to kill or disrupt any potential foreign organisms, and chemically ‘sound the alarm’, by attracting more white blood cells (leucocytes) to the affected area. To test whether this immune response was triggered by the presence of diamond, test samples made from diamond were placed in contact with neutrophils and the effects observed. It was found that adhesion was not increased, degranulation was not triggered, and reactive oxygen species and chemoattractants were not released [2,54,55]. In other words, the neutrophils were not activated by diamond.

Monocytes, another patrolling white blood cell, migrate to a site of invasion where they differentiate into macrophages—specialized scavenger cells, charged with disposing of all foreign material. Current data suggest that diamond particles do not activate the monocytes either [15]. Furthermore, macrophages were reported to uptake diamond particles without any detrimental consequences on their morphology, motility and viability. By contrast, macrophages exposed to silica crystals became immobile and died [2,56]. Subsequent studies indicated that diamond particle internalization by macrophages did not result in an inflammatory response [57].

**Figure 3.**
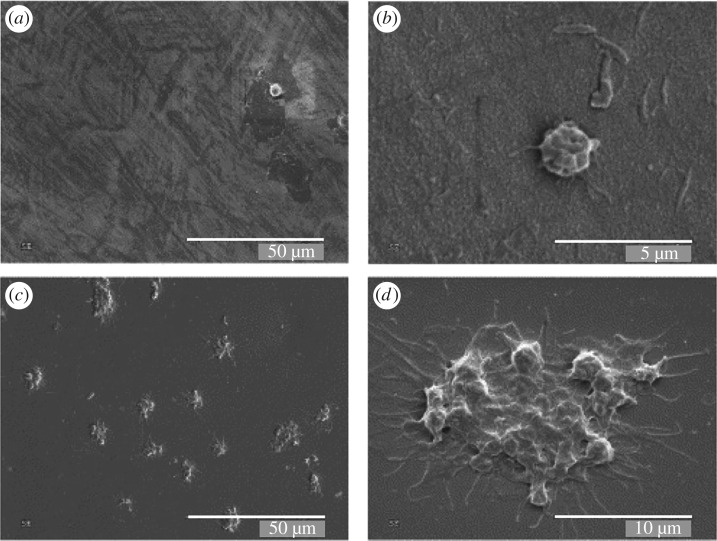
Platelet adherence to (*a*,*b*) diamond and (*c*,*d*) stainless steel. Very few platelets adhere on diamond, and even those that do so maintain a round morphology and do not attach and spread on the material. Scale bars (*a*,*c*) 50 µm and (*b*) 5 µm, (*d*) 10 µm. Modified from [62], and reproduced with permission.

Blood coagulation is a second (non-immune) branch of the body's defence. Its role is twofold: to limit the external loss of blood after an injury and to limit the internal invasion of foreign agents. However, in relation to implantable devices, coagulation can become extremely detrimental as it can isolate the implant behind a blood clot, induce stenosis (narrowing) of adjacent blood vessels and trigger hypoxia-activated inflammation. Regrettably, very few studies investigating the role of crystalline diamond in coagulation have been performed to date. However, for the very similar material, DLC, it was observed that fibrinogen (the main component of blood clots) adsorption, and platelet activation and aggregation are all low [58,59]. Moreover, subsequent studies showed that this low thrombogenic activity of DLC is dependent on the concentration of sp^3^ carbon states (diamond) [60,61]; in other words, higher diamond-like to graphite-like carbon ratios in the film result in reduced platelet adhesion. It should also be noted that the low fibrinogen adsorption is considered crucial for the low level of neutrophil activation [2]. One of the few studies [62] investigating crystalline diamond, rather than DLC, found that platelets could not adhere on diamond, as opposed to stainless steel where single platelets and clusters adhered, forming blood clots (figure 3). Recently, the development of artificial cardiovascular devices has been reported using UNCD coatings [63]. Preliminary studies by the US Company ADT (http://www.thindiamond.com/) showed that UNCD-coated valves for an artificial heart eliminated thrombus formation due to the extreme hydrophobicity of the coatings. This suggests that UNCD could provide low-wear and low-friction coatings, as well as being antithrombogenic.

Another important feature is that diamond particles do not induce haemolysis (the rupturing of red blood cells and the release of their contents into the surrounding fluids) [4]. This is a fundamental requirement if diamond is ever to be used as a coating for intravascular devices. While consistent *in vivo* research supports DLC's endothelial biocompatibility [64–66], the scientific and medical communities are still waiting for similar data to be reported for diamond.

Diamond doping is an essential feature in terms of bio-functionality, and boron is one of the most widely used dopants employed to change its electrical conductivity. Despite many boron compounds being potentially toxic, a number of *in vivo* as well as clinical studies have shown that boron-doped diamond (BDD) is highly biocompatible and integrates well with muscle and bone [1,67–69]. *In vitro* [14,24] and *in vivo* [70,71] research indicates that BDD is also biocompatible for neural tissue, supporting neuronal cell growth as well as tissue-electrode integration in the central nervous system (CNS). As described later (§4.2), *in vitro* BDD is indistinguishable from undoped diamond in supporting human neuronal cell cultures, even when cells are challenged with an oxidative insult [17]. Together, these results suggest that the boron atoms are locked into the diamond lattice and cannot leach out to affect nearby cells adversely, although long-term studies need to be performed to confirm if this remains true over periods of years or decades.

### 3.3. Limits of biocompatibility and caveats

Although diamond is bioinert, there are limits to its biocompatibility, at least in the case of nanoparticles. At very high concentrations (over 200 µg ml^−1^) NDPs were found to inhibit macrophage metabolic activity and proliferation rate [57]. Furthermore, high concentrations of NDPs were found to be toxic to aquatic life [72]. Interestingly, mammalian cells tolerate at least one order of magnitude more concentrated solutions of NDPs compared to aquatic crustaceans.

An interesting exception to diamond's general biocompatibility is its reported antibacterial properties. Mechanistic studies have found that different bacterial species show maximum sensitivity at particular NDP sizes [73], leading to the inference that specific interaction between the diamond nanoparticle and the bacterial wall are responsible for this effect. Other groups noted a correlation between NDP's surface termination and antimicrobial activity, with maximum effect observed for partially oxidized and negatively charged surfaces [74]. Diamond films have also shown bactericidal properties, especially when they are hydrogen-terminated and coated onto surfaces consisting of nanostructured needles, such as black silicon [75].

Collectively, these data confirm diamond's high biocompatibility and low cytotoxicity. They point towards the absence or very low level of inflammation and coagulation. As predicted, cellular accumulation of non-degradable particles can have a detrimental effect, which is dose-dependent. However, in the case of diamond, any toxicity can only be observed at significantly high doses, well above what would be necessary for medical treatments or investigations. Furthermore, the vast majority of medical, or medical-research-related procedures involve non-particle, macroscopic diamond devices for which no toxicity has been reported so far (see §4.1). Nevertheless, data from metal alloy and ceramic orthopaedic implants have raised some concerns regarding *in situ* generation of toxic nanoparticles, especially when significant mechanical wear is involved. In other materials, for instance titanium dioxide nanoparticles, an increase in reactive oxygen species formation has been observed, along with double-strand DNA breaking, inducing cell-cycle arrest [76]. This may partly be due to the fact that metallic, and to a lesser extent, ceramic nanoparticles are genotoxic, inducing irreversible DNA damage [77]. Cases of tissue necrosis produced as a consequence of such nanoparticle debris have also been reported [78]. With this in mind, a recent study compared UNCD particles with TiO_2_ particles in rat tissues. TiO_2_ particles induced necrosis and cell membrane damage accompanied by generation of reactive oxygen species both in lung and liver tissue. Conversely, none of these effects were observed in the case of UNCD nanoparticles [79].

It should be noted that, while diamond debris, most probably composed of NDPs, can be expected to be less genotoxic than metal and ceramic particles, this still needs to be accurately assessed by future studies. Notably, studies of DNA damage in human tissues are lacking. In embryonic stem cells, NDPs increased the expression level of DNA repair proteins (indicative of reversible DNA damage). This effect was more pronounced for oxidized NDPs, while being lower for all diamond samples tested compared with multiwalled carbon nanotubes [80]. Furthermore, the level of particle release from diamond implants has not been assessed yet, and this could be presumed to have the highest significance for orthopaedic applications.

## 4. Diamond as a substrate for cell culture and biomedical implants

Although diamond biocompatibility has been known for a long time, it was not until 1991 that a diamond-like material, in this case DLC, was used as a substrate for *in vitro* culture of mammalian cells (mouse fibroblasts and macrophages) [81]. More recently, crystalline diamond started to be considered, and in the last two decades many types of adherent cells have been successfully cultured on this substrate. These include mesenchymal stem cells [82,83], dental stem cells [84], cardiomyocytes (cardiac muscle cells) [85], neuronal stem cells [14,86–88], human-induced pluripotent stem cells (IPS) and IPS-derived neuronal progenitors [17], various types of neurons [24,29,43–45,89–91], osteoblasts [37,67,92–97], fibroblasts [16,89,98], macrophages [94,99,100] and epithelial cells [101,102]. While there is no question about the value of diamond as a culture substrate for adherent cells (in fact there is no cell type grown on any other material that cannot be cultured also on diamond), some controversies have arisen regarding the optimal culture conditions on diamond. These will be detailed in §4.2–4.4.

### 4.1. Diamond versus other biofriendly materials as a cell culture substrate

The first question which faced diamond researchers was: is diamond really superior to other routinely used substrates, or is it just another material on which cells can grow? It should be noted that because the procedures required for *in vitro* cell culture have been optimized for many decades on standard materials, any further improvements would probably be subtle. Even so, the value of diamond was readily apparent. When osteoblast (bone cells) and endothelial cells were cultured *in vitro*, they displayed similar high attachment and survival rates on plastic, glass and diamond substrates, but not on silicon [37]. Furthermore, cultured macrophages downregulated inflammatory cytokines on diamond compared to TCP [96], indicating that a diamond implant would activate the immune response to a lesser extent compared with other materials. Recent research seems to validate this claim, with experiments showing that UNCD-coated dental-type implants that were implanted *in vivo* in rat bones exhibited excellent osteointegration with no inflammatory response [103]. Furthermore, for a range of cell lines (epithelial and fibroblastic), diamond was found to be superior to silicon and platinum when the cell number, total cell area and cell spreading were quantified [104]. Direct comparison experiments showed that mouse fibroblasts also attached and proliferated significantly better on diamond compared to quartz [83], human epithelial cells adhered better on diamond versus glass [101,102], and neural stem cells proliferated and differentiated better on diamond versus TCP [86]. Other carbon-based materials, such as fullerene, carbon nanotubes and graphene, were also able to support the adhesion and growth of osteoblast and mesenchymal stem cells, and could support osteogenic differentiation without inducing DNA damage. In each case, diamond films and NDP had similar or superior cell-supportive properties [105].

### 4.2. Diamond surface characteristics

#### 4.2.1. Surface termination

Laboratory-grown CVD diamond is available with various surface terminations: *hydrogenated*, where the surface is covered with C–H bonds, and *oxygenated*, where the surface contains mainly carbon–oxygen bonds in a mixture of forms, such as ether (C–O–C), carbonyl (C=O) or hydroxyl (C–OH) groups [106]. O termination improves the hydrophilicity of the diamond surface and therefore is considered likely to enhance biocompatibility. Indeed, several studies have showed improved wettability on O-terminated compared with H-terminated UNCD, followed by superior cellular adherence and survival [101,102,107]. For instance, optimal adherence of human epithelial cells was found on oxygenated diamond surfaces, although pretreatment of the surface by protein adsorption mitigated the difference between O- and H-terminated surfaces [101,102]. For osteoblasts, the difference was so marked that patterned cell growth could be achieved simply by varying the type of surface termination across the diamond [95]. In a different study, it was found that O-terminated diamond supported the formation of bone extracellular matrix, with increased levels of calcium and phosphorus [84]. Furthermore, cell morphology followed the O-terminated region architecture. However, in at least one study, contrary results were reported. For the purpose of creating a wear-resistant bone prosthesis, researchers coated polished titanium with diamond to create a smooth surface, and then differentiated mesenchymal stem cells into osteoblasts. Here, robust cell adhesion and proliferation was found only on H-terminated diamond [107].

Nevertheless, on numerous other studies, O termination supported better biological applications. For example, adherence forces were measured for human fibroblasts on diamond and were found to increase by several times when the surface was modified from H to O termination [16]. Moreover, O termination of diamond favoured functionalization by bone morphogenic protein-2 (BMP2) adsorption, which promotes bone differentiation [92]. Similarly, neuronal cells could be successfully cultured on both H- and O-terminated surfaces, but a small increase in cell number was observed for O-terminated diamond [24]. One study found that O termination promotes oligodendrocyte differentiation without affecting neuronal differentiation [86]. Interestingly, for epithelial cell culture no phenotypical differences were found to be dependent on the type of diamond termination [101]. Although this could be a cell-type-specific phenomenon, more work is needed to establish a clear relation between diamond surface termination and differentiation biases.

H- or O-terminated laboratory-grown diamond can be further treated to alter the surface termination. Heating the sample in an atmosphere of the appropriate gases (e.g. Cl_2_, F_2_) allows the diamond surface to become Cl- or F-terminated [108,109]. This can also be done with plasma treatments using gas mixtures containing CF_4_, CCl_4_ or NH_3_ [110–112]. The latter produced aminated (NH_2_-terminations) diamond films, which may be candidates for later amide linkages to proteins or other biomolecules. Another common surface functionalization method is to use UV light along with a linker molecule containing a double bond at one end and a reactive group at the other. The UV radiation interacts with the double bond, which then displaces a surface hydrogen, resulting in the molecule being tethered to the diamond substrate by a covalent C–C bond. The reactive group (OH, NH_2_, etc.) at the free end of the molecule is then available for subsequent reactions [30]. Although many of these surface terminations have been investigated for their utility in electrochemistry or to attach biomolecules [33], only H- and O-terminated diamond have so far been used for cell culturing.

Finally, a recent study reported cell-specific preferences dependent on the sterilization method. It is presumed that various sterilization approaches can influence the type of diamond termination; and because the main goal of diamond is for it to be used in implantable devices, which require sterilization, this is highly relevant. The study found that cell density for cells cultured on diamond was maximal for samples sterilized by methods that increased hydrophilicity and that cortical neurons, one of the key target cells for brain implants, preferred autoclaved diamond [89].

#### 4.2.2. Surface topography

It is known that surface topography can significantly influence the differentiation and survival rates of various cells attached to it. For instance, it has been shown that mesenchymal stem cells can be stimulated to produce bone matrix simply by the nanoscale patterning of the polymethylmethacrylate (PMMA) substrate, even in the absence of osteogenic supplements [113]. The results were comparable with those from cells grown on a smooth PMMA surface cultured with standard osteogenic supplements. Furthermore, neural stem cells differentiated better, but proliferated less, on micro-patterned silicon versus flat Si surfaces, and these processes were dependent on mitogen-activated protein kinase (MAPK) signalling [114]. In the case of diamond, discrete topography can be produced by various methods, including controlling the size of the crystals in the polycrystalline structure by varying the CVD gases and conditions, and modifying the roughness of the wafer on which the diamond film is deposited. When it comes to cell preferences for the morphology of the polycrystalline diamond (PCD) surface, apparently smaller is better. Fibroblasts, osteoblasts and neural cancer cell lines attached, proliferated and had enhanced cellular functions on NCD compared with MCD [16,37], and on NCD deposited on low-roughness substrates compared with that on high-roughness substrates [70]. In the case of human neural progenitors, optimal cell support was provided by the MCD structure with the smallest crystals (up to 1 µm) [17]. These findings were confirmed in a different study [14], where NCD crystallites were grown on microstructured carbon nanotubes (with the diamond crystals replicating the underlying microstructure). In this case also, optimal support for neuronal progenitors was observed for 1-µm-scale diamond structures. Nanoscale topography on an NCD surface was also reported to promote superior osteoblast differentiation compared with that seen on TCP [115]. Another study by Tong *et al*. [116] concluded that diamond surface roughness was critical for growth of healthy rat cortical neurons, with the best results observed on surfaces with a roughness of about 20 nm. These authors also suggest that there may be a narrow window of surface roughness for optimum neuronal adhesion.

Despite the variation in the reports, the consensus seems to be that, in general, cells prefer to grow on diamond surface structures and morphologies with sizes of the order of 100 nm to 1 µm. It is very likely that future research will bring critical insights into the mechanism by which topography influences cellular behaviour. Diamond will no doubt have an important role to play in this research due to the ease by which it can be patterned.

### 4.3. Diamond protein coating

A protein coating, acting as a bioactive interface onto which the cells can attach with their own matrix-anchoring proteins, as they would in a living tissue, is commonly used when mammalian cells are cultured on an artificial substrate. The requirement for this protein layer, as well as the specific protein formulation used, is cell-type dependent. An apparent reduced requirement for this protein layer was observed for various cellular applications employing diamond materials. This led to an interesting debate on whether a protein coating is necessary at all when diamond is used as a substrate. First, it was observed that human fibroblasts [98] and mouse hippocampal neurons [90] can be cultured on nanodiamond films without protein coating. This observation is significant in the context that fibroblasts can routinely be cultured without a protein coating on TCP but not on standard glass, while neurons require a coating on both. While these ‘protein free’ culture methods on diamond have not been universally accepted, Thalhammer *et al*. [44] reported superior adhesion of neurons on various inorganic substrates coated with protein-free NDPs. These reports have subsequently been disputed by Ojovan *et al*. [45], who suggested that the previous findings may have been confounded by adsorption onto the diamond substrate of other proteins present in the culture media used to sustain the cells. As all media formulations used to culture neurons contain a proteic component, it is very likely that future studies will face significant challenges in accurately delineating the need for an external protein interface between diamond and neurons.

Finally, it should be noted that while some groups adopt a minimalistic approach, trying to reduce or eliminate the protein coating, others have embraced diamond's ability to readily adsorb protein. Thus, osteogenic differentiation could be enhanced by adsorbing BMP2 on diamond prior to cell culture [92,117,118], while in a different study, researchers were able to inhibit an inflammatory response in macrophages by pretreating diamond with collagen and dexamethasone [99].

### 4.4. Diamond for bone repair

**Figure 4.**
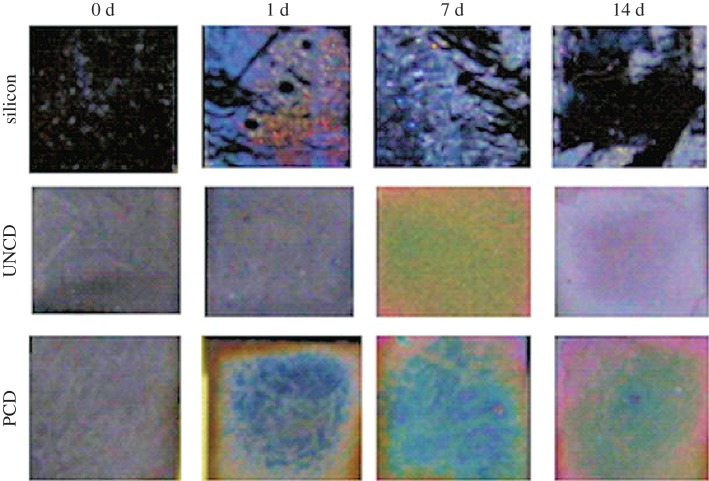
Images showing that artificial body fluids form spontaneous apatite deposits after several days in contact with various substrates. After the first day, only a thin deposit formed on silicon, which subsequently peeled off. By contrast, with UNCD and MCD (labelled PCD in the figure) strong deposits were formed on the diamond surface which survived for 14 days. Reproduced with permission from [120]. (Online version in colour.)

Bone was one of the first target tissues for which a diamond implant was considered. There is significant scope in coating classical joint and bone implants with crystalline diamond for the purpose of increasing biocompatibility and adhesion, and reducing adverse reactivity, inflammation, erosion and release of metal nanoparticles into the bloodstream. The challenge here is to optimize the CVD process on standard implant alloys, as often the diamond deposition process is poor on metals. Significant progress has been made for deposition on titanium, cobalt–chromium and even steel substrates (see the review by Catledge *et al.* [119]). Early reports have been very encouraging, as researchers found that coating titanium alloys with diamond films increased erosion resistance while providing excellent biocompatibility [67]. This observed biocompatibility was later confirmed when fibroblast cell lines and human bone-marrow cells grown on diamond displayed superior viability, proliferation, alkaline phosphatase (ALP) activity and extracellular matrix mineralization and deposition, compared to those grown on TCP [37,96]. Similarly, in an elegant experiment, silicon and crystalline diamond were placed in contact with ‘simulated body fluid’ for up to 14 days, and the authors observed superior spontaneous deposition of apatite (a precursor of hydroxyapatite—the major component of bone matrix) on NCD [120] (figure 4). Consistent with previous reports, increased proliferation and superior bone differentiation was observed for NCD versus MCD [97], and superior osteoblast function (including collagen production, calcium deposition and ALP activity) was observed for O termination compared to H termination [84,121]. Furthermore, osteoblasts could be patterned by selective O and H termination of the diamond surface, with the cells colonizing preferentially the O-terminated regions, while the H-terminated regions could only support cellular growth when cells were seeded at high densities [95]. In a similar study, cells preferentially arranged themselves on the O-terminated surface [84].

Interestingly, when BDD was used, differences could be observed for bone differentiation compared to undoped diamond. For instance, one study reported superior cell adhesion and improved collagen and vinculin production for osteoblasts plated on diamond with moderate levels of boron doping, compared both with heavily doped and undoped diamond [93]. In a recent study, increased hydroxyapatite deposition was observed on BDD, and the authors speculated that this might be connected to electrical conductivity [122]. Furthermore, *in vivo* studies reported that diamond-coated implants showed high biocompatibility, good osteointegration and an increased ability to promote bone growth compared with standard surgical materials [1,68].

Notably, diamond can be functionalized by simple adsorption of bioactive reagents, which can then induce a biological response. For example, NCD functionalized by BMP2 (an inducer of osteogenic differentiation) activated the expression of osteoblast markers in human mesenchymal cells, indicating that BMP2 is functional when adsorbed onto diamond. Furthermore, reports of NDPs functionalized with angiotensin (a factor that stimulates new blood vessel generation) suggest they could be used to promote repair of damaged or degraded bone [92,117,123].

Together, these reports establish diamond as a strong material candidate for bone repair, and it is likely that dental implants will lead the front of innovation here [103]. However, for the more challenging (and maybe even more significant) joint repair, more research involving cartilage cells and tissue is still required.

### 4.5. Diamond for brain research

**Figure 5.**
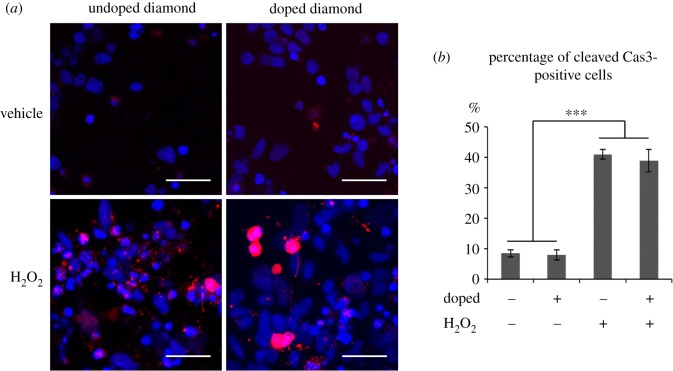
(*a*) Fluorescence microscopy of human cortical neurons plated on BDD or undoped diamond and challenged with H_2_O_2_, an oxidative stress inducer. The blue regions show cells stained with Hoecsht stain and identify the cell nuclei, while the red regions are stained with cleaved caspase 3, a marker of apoptosis (cell death). Scale bars, 25 µm. (*b*) Quantification of cells stained for cleaved caspase 3. Error bars = standard error of the mean, ***p < 0.001 (between H_2_O_2_ challenged and unchallenged samples). The bar-chart indicates that BDD shows similar biocompatibility to undoped diamond. Even when challenged with oxidative stress, the cellular response is indistinguishable between the two substrates. Thus, the presence of B in the diamond appears to be non-toxic as far as cell growth is concerned. Reproduced from [17] under CC-BY licence. (Online version in colour.)

As described above, mammalian neurons, including human ones, can be successfully cultured on diamond substrates. The optimal culture conditions and surface characteristics have been investigated on undoped diamond and BDD, the latter of which can function as an electrical conductor. Importantly, those two diamond-based materials were shown to have indistinguishable biocompatibility in neuronal cultures (figure 5) [14,17,71,93,124]. A major breakthrough was achieved when human cortical neurons were cultured long term (over 90 days) on diamond substrates, with cells showing phenotypically mature synapses [17]. This is essential both for prospective studies envisaging brain implants (which must remain functional for many years *in vivo*), as well as for performing complex studies on artificial neuronal networks *in vitro*.

**Figure 6.**
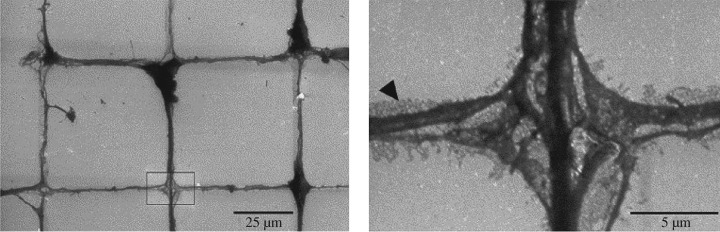
Patterned artificial neuronal networks, following a pre-stamped laminin grid on a diamond substrate. Reproduced with permission from [29].

Progress in investigations of artificial neuronal networks has been made recently by various studies showing directed neuronal growth on diamond, where the neurons have been ‘persuaded’ to grow preferentially in certain areas. These patterning studies are especially encouraging, considering that a range of different methods have proved effective. Thus, neurons have been patterned by contact micro-printing [29] as shown in figure 6, inkjet and laser patterning [24], selective seeding of NDPs [43] and diamond deposition on patterned carbon nanotubes [14]. Further studies showed that neuronal growth on various diamond and DLC substrates can be manipulated by a combination of laminin coating and surface termination [125–127]. Similarly, neuroblastoma cells have also been preferentially grown on UNCD patterned with O, F or H terminations, and this may lead to new routes to studying neuronal cancer [128].

Electrical activity of cultured neurons on diamond was demonstrated by the patch-clamping technique [17,44]. In a similar way, the signalling activity of cromaffin cells could be measured *through* a diamond electrode [129]. Although cromaffin cells are not neurons, they share structural similarities with sympathetic neurons, importantly sending their signals to the CNS via neurotransmitters released through synaptic vesicles. Thus, the measured activity of cromaffin cells can be equated to neuronal activity. Both these examples show that neurons and neuron-like cells cultured on diamond *in vitro* remain alive and healthy, and crucially are able to function while sitting on a diamond plate in a cell-culture dish. It is perhaps worth noting that cellular electrical signals can also be recorded on BDD electrodes from non-neural cells, such as cardiomyocytes [85], thus expanding the range of potential applications for these types of electrodes.

The ultimate goal of diamond biomedical research has always been the production of safe and reliable neural implants, and it is only in the last few years that scientific publications on this topic have started to appear. An excellent recent review of diamond for neuronal interfacing is available in [69]. The key point is whether diamond implants cause less inflammation and scarring than conventional materials, and reduce the formation of fibrotic capsules—a natural defence mechanism against foreign bodies, which risks electrically insulating the probe from its intended target. Some early evidence for this came when NCD implants placed on the retina of rabbits were reported to be well tolerated with minimal inflammation [10]. More recently, it was shown that fibrotic capsule formation and tissue reaction both decreased in the case of implants coated in NCD compared to ones made from medical-grade titanium or silicon [100]. Similar results were obtained when BDD and nitrogen-doped diamond (NDD) were used, with reduced fibrosis as well as reduced acute and chronic inflammatory response compared to silicone [69]. In addition, another recent study reported further reduction in encapsulation and inflammation for BDD compared to undoped diamond [124], although these latter findings await confirmation.

**Figure 7.**
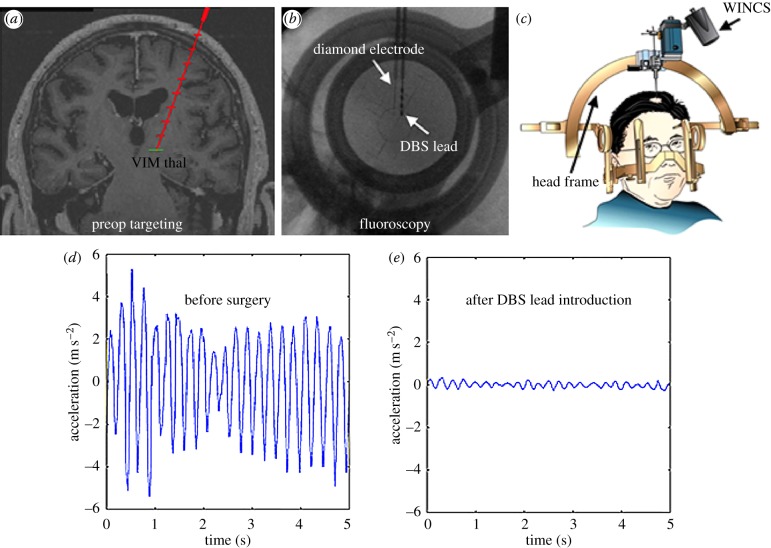
A patient treated for tremors using a BDD probe. (*a*) Brain computer tomography scan showing the predicted implant trajectory, (*b*) macroscopic image of the surgical lead and diamond probe, (*c*) surgical frame stabilizer, (*d,e*) accelerometry plots of the affected hand before and after the implant, showing decreased tremor. Reproduced from [135] under CC-BY licence. (Online version in colour.)

When diamond functionality was tested, ganglion cells from explanted rat retina could be stimulated through an NDD electrode, as shown by the action potentials measured via patch clamping [130]. Subsequently, two methods have been proposed to produce diamond-only electrode arrays for retinal prostheses [131,132], and it is conceivable that the same technology could be applied for stimulating other parts of the CNS. Furthermore, low impedance, low background noise and a large potential window have been reported for BDD electrodes. In one of the oldest studies of this type, BDD was implanted in the auditory cortex of a live guinea pig, and neuronal sound-dependent stimulations were recorded [133]. Similarly, UNCD thin film-covered microchips were successfully implanted on the retina of rabbits [10]. More recently, BDD was used to record the local field potential from whole-embryo preparations of mouse hind-brain and spinal cord [134]. Furthermore, when BDD electrodes were implanted in a rat cortex, it was shown that they generated reduced magnetic resonance imaging (MRI) interference compared to metal electrodes [71]. This offers the exciting prospect of using MRI as a tool to position diamond implants more accurately in sensitive locations, such as the brain, or to use dynamic MRI to visualize the effects of neural stimulation via the implant in real time—both of which are very difficult to achieve with metal implants. Recently, BDD electrodes have been used for deep brain stimulation, by implantation in the thalamus of patients suffering from essential tremor (figure 7) [135]. A similar procedure could be used to treat Parkinson's-related tremor.

## 5. Conclusion

The development of CVD technology in the past two decades has opened the door for the use of crystalline diamond films and nanoparticles for a range of biomedical applications. Of note is the ability to control material characteristics, such as electrical conductivity (through boron and nitrogen doping), surface termination, crystal size and, very importantly, to micro-pattern the diamond surface by a range of methods into biologically significant structures. Decades of research have established diamond as one of the most biocompatible substrates known, thus adding one more property to the long list of superlative characteristics of this material. The range of potential uses for diamond in medicine is large, including: blood vessel stents, microprobes, artificial joint components and coatings for devices that would facilitate bone growth or repair. Bioinert, but electrically conducting diamond films can be used to make neural implants that can pass signals to and from neurons in the brain, spine or peripheral nervous system (hands, legs, fingers), with minimal inflammation, scarring or signal loss. This offers the possibility of neural implants with lifetimes of decades—potentially longer than that of the patient—reducing the need for repeat surgeries and their associated risks, complications and costs. Such advances could usher in a new era in permanently implanted diamond electrodes for treatments of a range of neurological diseases, such as epilepsy, Parkinson's, paralysis and even stroke.

Importantly, we are now at a stage where artificial neuronal network technology is beginning to mature. Two-dimensional networks of animal or human neurons can now be created in the lab on a diamond substrate, where the neurons follow predesigned patterns and grids, joining at crossing points, and passing electrical signals between each other and through the network as they would in a living body. This is only possible because of the long lifetime of the neurons on diamond compared to other substrates, and made simpler by using the BDD substrate itself as a means to send electrical stimuli into and out of the neural network. Such two-dimensional neural networks may provide invaluable models with which neuroscientists could simulate the more complex three-dimensional network of a brain. Another benefit of these neural networks is that significant research that would be too complicated, too expensive or too ethically challenging to be carried out *in vivo* can now be performed *in vitro*, in well-defined, reproducible conditions. These would include genetic studies, age-related and degenerative disease studies, investigation of neurotoxins, as well as performing high-throughput screens for neurological drugs.

The future for diamond-based substrates and implants certainly looks promising; however, we should sound a note of caution. Although it is relatively straightforward to obtain funding to develop end-user applications, such as the multinational projects mentioned earlier, supported by governments and biomedical companies to make artificial retinas, bionic eyes and other neuronal interfaces, there is the danger that these may underperform due to a lack of understanding of the fundamental interface between diamond and cells. Obtaining funding to study these basic questions is much more difficult, but without it researchers may find themselves trying to ‘run before they can walk’. If the early bioimplants prove to have disappointing performance due to unexpected problems arising at the diamond–cell interface, then this may prejudice the entire case for using diamond in these applications. Hence, the urgent need for governments, medical agencies, charities and industry to fund the basic science underpinning the devices, as well as the development of the devices themselves.

While only time can tell how sparkling the prospects for diamond in the medical field might be, diamond is a material that biologists, medical researchers and practitioners are going to become increasingly familiar with.

## Data accessibility

This article has no additional data.
